# Parental age at birth and biomarkers of fecundity in young Danish men

**DOI:** 10.1111/andr.13536

**Published:** 2023-09-26

**Authors:** Andreas Ernst, Nis Brix, Anne Gaml‐Sørensen, Linn Håkonsen Arendt, Gunnar Toft, Sandra Søgaard Tøttenborg, Karin Søring Hougaard, Jens Peter Ellekilde Bonde, Cecilia Høst Ramlau‐Hansen

**Affiliations:** ^1^ Research Unit for Epidemiology, Department of Public Health Aarhus University Aarhus Denmark; ^2^ Department of Urology Aarhus University Hospital Aarhus Denmark; ^3^ Department of Clinical Genetics Aarhus University Hospital Aarhus Denmark; ^4^ Steno Diabetes Center Aarhus Aarhus University Hospital Aarhus Denmark; ^5^ Department of Occupational and Environmental Medicine Copenhagen University Hospital‐Bispebjerg and Frederiksberg Copenhagen Denmark; ^6^ Department of Public Health University of Copenhagen Copenhagen Denmark; ^7^ National Research Centre for the Working Environment Copenhagen Denmark

**Keywords:** cohort study, epidemiology, parental age, paternal factors, reproductive health, semen quality

## Abstract

**Background:**

High parental age is associated with adverse birth and genetic outcomes, but little is known about fecundity in male offspring.

**Objectives:**

We investigated if high parental age at birth was associated with biomarkers of male fecundity in a large population‐based sample of young men.

**Materials and methods:**

We conducted a study of 1057 men from the Fetal Programming of Semen Quality (FEPOS) cohort, a sub‐cohort of sons born 1998–2000 into the Danish National Birth Cohort. Semen characteristics and reproductive hormone concentrations were measured in samples provided by the men 2017–2019. Testis volume was determined by self‐measurement. Data on the parental age was drawn from registers. Adjusted relative difference in percentage with 95% confidence intervals were estimated for each outcome according to pre‐specified maternal and paternal age groups (< 30 (reference), 30–34 and ≥ 35) as well as for combinations of parental age groups, using multivariable negative binomial regression models.

**Results:**

We did not observe consistent associations between parental age and biomarkers of fecundity, although sons of mothers ≥ 35 years had lower sperm concentration (−15% (95% CI: −30, 3)) and total sperm count (−10% (95% CI: −25, 9)). The analysis with parental age combinations showed lower sperm concentration with high age of the parents (both ≥ 35 years: −27%, 95% CI: −40, −19) when compared to the reference where both parents were below 30 years.

**Discussion and conclusion:**

We found no strong association between higher parental age and biomarkers of fecundity in young men. However, we cannot exclude poorer semen characteristics in sons born by older mothers or with high age of both parents.

## 1 INTRODUCTION

Infertility represents a major global health problem that affects up to 15% of couples worldwide.^1^ Male factors are involved in up to 50% of the cases,^2^ of which impaired semen quality is the most important.^3^, ^4^, ^5^ Infertility in couples, but also poor male fecundity by itself, have links to several long‐term comorbidities.^6^, ^7^

In the past century, family planning, use of contraception and availability of fertility treatment options have been suggested to increase subfecundity at the population level.^8^ Family planning has been followed by a delay in parenthood from the early 1970s in Western countries.^9^ The demographic shift toward postponed parenthood has received increasing attention due to potential adverse biological effects on the health of the next generations.^10^ Studies consistently show increased risk of several adverse birth outcomes and chromosomal abnormalities in children born by older mothers.^11^, ^12^ Further, also higher paternal age, especially above 45 years, has been associated with adverse birth outcomes, offspring genetic disorders and genital malformations.^13^, ^14^ The proposed underlying mechanisms include accumulation of hazardous environmental exposures, changes in fetal–placental homeostasis, mutations in spermatozoa, aneuploidies due to non‐disjunction during meiosis in the ovum and epigenetic deviations.^15^, ^16^, ^17^

Similar mechanisms have potential to affect the reproductive health in the sons, which we would expect to lower the semen quality and disrupt hormonal balances. The hypothesis is supported by earlier studies showing that the hypothalamic–pituitary–gonadal‐axis (HPG‐axis) as well as the development and functioning of the testes are sensitive to external stimuli.^18^, ^19^ However, Priskorn et al. published in 2014 a Danish register‐based cohort study with 10,965 men reporting no association between parental age and semen quality in the sons.^20^ The study's strengths include its large size and wide exposure contrast, but it was based on men referred due to partnership infertility over a period of 40 years (1960–2000), where standardization and quality control of semen analyses were lacking. Further restraints included lack of information on confounders and additional biomarkers of fecundity.

We aimed to investigate if high parental age at birth was associated with fecundity in a population‐based sample of young men assessed by various biomarkers: semen characteristics, testis volume, and reproductive hormone concentrations.

## 2 MATERIALS AND METHODS

Our study is based on data from the male‐offspring sub‐cohort, FEPOS, nested within the Danish National Birth Cohort (DNBC, primarily of Caucasian origin). A full‐length cohort description is found elsewhere.^5^ In short, FEPOS was established in March 2017 and data collection was finalized in December 2019. The eligibility criteria for participation were: (1) information from the first two DNBC pregnancy interviews scheduled at gestational week 12 and 30 available, (2) a gestational blood sample in the DNBC biobank, (3) older than 18 years and 9 months at recruitment, and (4) living in proximity of the research clinics in Copenhagen or Aarhus. In total, 21,623 sons full‐filled the criteria of whom 5,697 were sampled in a consecutive manner for invitation. In the invitation letter, the sons were encouraged to decline participation if they: (1) had undergone sterilization, (2) had received chemotherapy, or (3) for any reason only had one or no testes in the scrotum. The follow‐up regime for those who agreed to participate and provided informed consent included: (1) an online comprehensive questionnaire on health behavior (2) a semen and a blood sample, and (3) a clinical visit. In total, 1,057 sons completed the entire follow‐up (Figure S1).

### 2.1 Parental age

A unique 10‐digit identifier (Civil Registration Number (CNR)) is assigned to Danish residents at birth or immigration and enables individual level record linkage across registers in the health care system. The identifier is composed of the birth date followed by a unique 4‐digit sex‐specific code.

Information on maternal age on the exact delivery date of the index child was retrieved from the Danish Medical Birth Register (DMBR) and categorized: < 30 (reference, *n* = 443), 30–34 (*n* = 429), and ≥ 35 (*n* = 185). The maternal CRN was used to identify the father of the index child through linkage in the Civil Registration System. Subsequently, paternal age at delivery of the index child was calculated by subtracting the delivery date from the paternal birth date available from his CRN. Next, paternal age was categorized: < 30 (reference, *n* = 290), 30–34 (*n* = 391), and ≥ 35 (*n* = 367). A total of nine fathers had missing information on age, making up our final study populations to 1,057 and 1,048 sons for the maternal and paternal analyses.

### 2.2 Semen characteristics and testis volume

The semen samples were collected at home or at the research clinic in pre‐weighed containers. A sample kit was provided together with detailed instructions. Semen analyses were handled by expert biomedical laboratory technicians blinded to the exposure, conducted according to WHO 2010 standards.^21^ An internal quality control showed that the semen analyses met international standards.^5^

Semen volume was derived by weighing of the sample (1 g = 1 mL). Sperm concentration was derived by manual counting of two aliquots using a BLAUBRAND Improved Neubauer Hemocytometer (BRAND, Wertheim, Germany) diluted with NaHCO_3_ resolution according to the concentration. The diluted aliquots was transferred to each chamber of the hemocytometer and rested in a humid chamber for 10 min prior to counting. Total sperm count was derived as the product of semen volume and concentration. Sperm motility was derived by counting the percentage distribution of progressive motile (PR), non‐progressive motile (NP), and immotile (IM) in 200 spermatozoa within each of the two fresh drops of semen. The sum of the percentage of NP and IM was used as outcome to ensure optimal model fit. Sperm morphology was derived as the proportion of normal sperm cells analyzed at the Reproductive Medicine Centre, Skåne University Hospital, Sweden.

The volume of each testis was self‐measured at the clinical visit using a Prader Orchidometer (Bayer AG, Leverkusen, Germany), previously shown valid in comparison to an expert examination.^22^ The average testis volume was used as outcome.

### 2.3 Reproductive hormones

A non‐fasting venous blood sample was collected at the clinical visit. The following reproductive hormone concentrations were measured at Department of Clinical Biochemistry, Aarhus University Hospital, Denmark according to international standards and guidelines: follicle stimulating hormone (FSH), luteinizing hormone (LH), testosterone, estradiol, and sex hormone‐binding globulin (SHBG).^5^ SHBG, FSH, and LH were measured using immunoassays (Cobas 8000 e602; Roche Diagnostics, Mannheim, Germany) with CVs of 2.5%–2.8%, 0.7%,–1.2%, and 1.1%–1.7%, respectively. Testosterone and estradiol were measured using liquid chromatography‐tandem mass spectrometry (LC–MS/MS). The limits of detection (LOD) were: FSH: 0.1 IU/L (*n* < 5); LH: 0.1 IU/L (*n* < 5); and estradiol: 15 pmol/L (*n* = 87). All participants had concentrations above the LOD for SHBG and testosterone. Hormone concentrations below the LOD were replaced by the LOD divided by √2. We derived the free androgen index (FAI) as: (total testosterone/SHBG) × 100%.

### 2.4 Covariates

We identified potential confounders by drawing directed acyclic graphs based on a literature review. The maternal analyses were adjusted for the highest parental socioeconomic status (defined by ISCO‐88 and ISCED), maternal first trimester smoking, maternal pre‐pregnancy body mass index (BMI), parity, and paternal age. The paternal analyses were adjusted for the highest parental socioeconomic status, paternal smoking, and maternal age. Information on the covariates was available from the DNBC interviews except for the parity available in DMBR.

To improve precision, we also adjusted the analyses of the semen characteristics for abstinence time, place of sample collection, and spillage as these parameters were considered strongly correlated to the outcomes. However, we excluded participants with (1) spillage at collection from the analyses of semen volume and total sperm count (*n* = 182), and (2) azoospermia from the analyses of motility and morphology (*n* = 17). The interval from collection to analysis of the semen sample was further included for motility analyses. Only abstinence time was included in the analysis of testis volume. Only timing of blood sampling was included in the analyses of reproductive hormone concentrations. See Table 1 for further details on the covariates.

**TABLE 1:** Baseline characteristics according to categories of maternal age at birth, *n* = 1057, FEPOS, Denmark, 1998–2019.

	<30 years^a^ *N* = 443	30–34 years *N* = 429	≥ 35 years *N* = 185	Missings, *N*
Maternal pre‐pregnancy BMI (kg/m^2^), *n* (%)^b^				25
Underweight	30 (6.9)	21 (5.0)	13 (7.3)	
Normal	304 (70.4)	315 (74.8)	138 (77.1)	
Overweight	77 (17.8)	62 (14.7)	25 (14.0)	
Obese	21 (4.9)	23 (5.5)	< 5 (.)	
Maternal smoking during 1. trimester, *n* (%)				0
Non‐smoker	323 (72.9)	338 (78.8)	153 (82.7)	
0–10 cigarettes per day	102 (23.0)	80 (18.6)	23 (12.4)	
> 10 cigarettes per day	18 (4.1)	11 (2.6)	9 (4.9)	
Maternal age at birth (years), mean (SD)	27.1 (2.1)	32.4 (1.4)	37.2 (1.9)	0
Paternal age at birth (years), mean (SD)	30.0 (4.4)	34.6 (4.4)	38.6 (5.4)	9
Paternal smoking, *n* (%)				0
No	315 (71.0)	301 (70.2)	134 (72.4)	
Yes	128 (29.0)	128 (29.8)	51 (27.6)	
Parity, *n* (%)				>21
1. Child	270 (62.4)	160 (38.3)	38 (20.7)	
2. or more child	163 (37.6)	258 (61.7)	<147 (< 79.5)	
Highest parental social class, *n* (%)				0
High‐grade professional	99 (22.3)	176 (41.0)	>87 (47.0)	
Low‐grade professional	137 (30.9)	146 (34.0)	67 (36.2)	
Skilled or unskilled worker	171 (38.6)	97 (22.6)	31 (16.8)	
Student or economically inactive	36 (8.1)	10 (2.3)	< 5 (.)	
Time to pregnancy, *n* (%)				6
< 6 months	<289 (< 65.2)	<254 (< 59.2)	<94 (< 50.8)	
6–12 months	45 (10.3)	40 (9.3)	22 (12.0)	
> 12 months or MAR^c^	36 (8.2)	72 (16.8)	30 (16.3)	
Unplanned	73 (16.6)	63 (14.7)	39 (21.2)	
Place of semen sample, *n* (%)				10
Home	56 (12.8)	55 (12.9)	27 (14.8)	
Clinic	381 (87.2)	<374 (87.2)	<158 (85.4)	
Abstinence time, *n* (%)				5
< 2 days	146 (33.0)	152 (35.5)	68 (37.4)	
2–3 days	<205 (46.3)	<205 (47.8)	<84 (45.4)	
> 3 days	92 (20.8)	72 (16.8)	33 (18.1)	
Spillage, *n* (%)				9
No	367 (83.8)	<356 (83.0)	<147 (79.5)	
Yes	71 (16.2)	73 (17.1)	38 (20.9)	
Interval from ejaculation to analysis, *n* (%)				12
0–60 min	368 (84.2)	<356 (83.0)	<155 (83.8)	
> 60 min	69 (15.8)	73 (17.1)	30 (16.5)	
Time blood sampling, *n* (%)				11
Morning < 12 PM	157 (36.0)	155 (36.2)	65 (35.7)	
Afternoon 12–18 PM	232 (53.2)	<233 (54.3)	<97 (52.4)	
Evening > 18 PM	47 (10.8)	41 (9.6)	23 (12.6)	

Abbreviations: AAM, age at menarche; BMI, body‐mass index; ICSI, Intracytoplasmic sperm injection; IVF, In Vitro Fertilization; MAR, medically assisted reproduction; SD, standard deviation.

^a^ Due to local data regulations, it is not allowed to report smaller numbers than five. Thus, numbers have been changed to mask the numbers smaller than five.

^b^ The percentage distribution for the levels of each specific baseline characteristic within each age category.

^c^ In total, 67 children were conceived by MAR of whom IVF/ICSI accounts for 26 children.

### 2.5 Statistical analyses

Baseline characteristics were presented as percentages or as means with standard deviations according to parental age categories. The medians and 5th–95th pseudo percentiles (percentile average value of the five observations nearest to the given percentile to comply with the General Data Protection Regulation, GDPR) were calculated for each outcome. We accounted for the overdispersed distribution of the outcomes by choosing a negative binomial regression model, which fitted the data favorably. To visually present the functional form of the crude associations, we modeled the semen characteristics, testis volume, and reproductive hormone concentrations as flexible functions of parental age included as restricted cubic splines with three knots located at the 10‐, 50‐, and 90‐percentiles (maternal age: 24.8, 30.6, and 36.7 years; paternal age: 26.7, 32.4, and 40.9 years).

In the main analyses, we estimated adjusted mean ratios with 95% confidence interval (95% CI) for each of the outcomes as a function of maternal and paternal age categories with < 30 years as reference. In these models, categorical explanatory variables (specific sets of confounders and precision variables) were included as indicators, whereas mutual adjustment for maternal or paternal age was performed with second‐order polynomials. To ease interpretation of the results, the ratios were converted to relative differences in percentage: (ratio − 1) × 100%.

We conducted the following sub‐analyses. First, we subdivided the oldest paternal age group into two groups allowing us to look for specific patterns among the sons born by the oldest fathers (≥ 45 years, *n* = 37). We were unable to conduct a similar sub‐analysis for maternal age due to a more narrow age range and very few mothers with a higher age at giving birth. Second, we performed an analysis to examine the association between combinations of maternal and paternal age categories and sperm concentration, as sperm concentration has been the factor most strongly associated with probability of conception.^3^

Selection weights were included in all analyses to account for potential selective non‐participation in FEPOS.^23^ The weights were derived as the inverse probability of participation based on a multivariable logistic regression model including a range of a priori chosen potential predictors of participation. Robust standard errors were applied to account for clustering of siblings and the inclusion of selection weights. To check for violations of the model assumptions, the observed distributions of the outcomes were compared against the model‐based distribution using *Q*−*Q*‐plots. Further, the standardized deviance residuals were compared against the model‐based predictions. The plots did not give rise to any concerns (data not shown). Analyses were performed in Stata MP 17 (StataCorp, College Station, TX, USA).

### 2.6 Ethical approvals

Informed consent was obtained from each participant included in the study. The establishment of the FEPOS cohort was approved by the Scientific Research Ethics Committee for Copenhagen and Frederiksberg (No. H‐16015857) and the Danish Data Protection Agency (No. 2012‐58‐0004, 231). Moreover, recruitment and data collection in the FEPOS cohort were permitted by the Steering Committee of the DNBC (Ref. no. 2016‐08, 2018‐09). This study was performed according to the Declaration of Helsinki.

## 3 RESULTS

The median age at participation in the clinical examination was 19.2 years (interquartile range: 19.0–19.4). In the study population, the mean maternal and paternal ages at birth were 31.0 (SD: 4.2, pseudo range: 19.7–42.2) and 33.4 years (SD: 5.6, pseudo range: 21.4–56.1). The oldest parental age groups were, in comparison to the reference groups, of higher socioeconomic status, less likely to smoke and more often of higher parity. The precision variables were evenly distributed (Table 1 and Table S1). The crude distribution of semen characteristics, testis volume and reproductive hormones can be found in Table S2.

### 3.1 Main analyses: maternal age

The spline plots for maternal age showed an inverted U‐shape for sperm concentration and total sperm count but a U‐shape for morphology and testis volume (Figure 1A). The curve for testosterone was close to flat, while a U‐shaped relation was indicated for estradiol. In the adjusted analyses, the findings according to maternal age categories were not consistent across semen characteristics (Figure 2A and Table S3). However, sperm concentration and total sperm count tended to be lower with age, especially among sons of mothers ≥ 35 years (concentration: −15% (95% CI: −30, 3); count: −10% (95% CI: −25, 9)). FSH, LH and estradiol tended to be higher with age, especially among sons of mothers ≥ 35 years (FSH: 14% (95% CI: 0, 29); LH: 9% (95% CI: 0, 18); and estradiol: 7% (95% CI: −5, 21)).

FIGURE 1(A) Semen characteristics, testis volume, and reproductive hormones as spline functions of maternal age at birth, *n* = 1057, FEPOS, Denmark, 19982019. Solid lines represent crude estimates with dashed lines representing 95% confidence intervals. (B) Semen characteristics, testis volume and reproductive hormones as spline functions of paternal age at birth, *n* = 1048, FEPOS, Denmark, 1998–2019. Solid lines represent crude estimates with dashed lines representing 95% confidence intervals.Abbreviations: FSH, follicle stimulating hormone; LH, luteinizing hormone; SHBG, sex‐hormone binding globulin.

**Figure:**
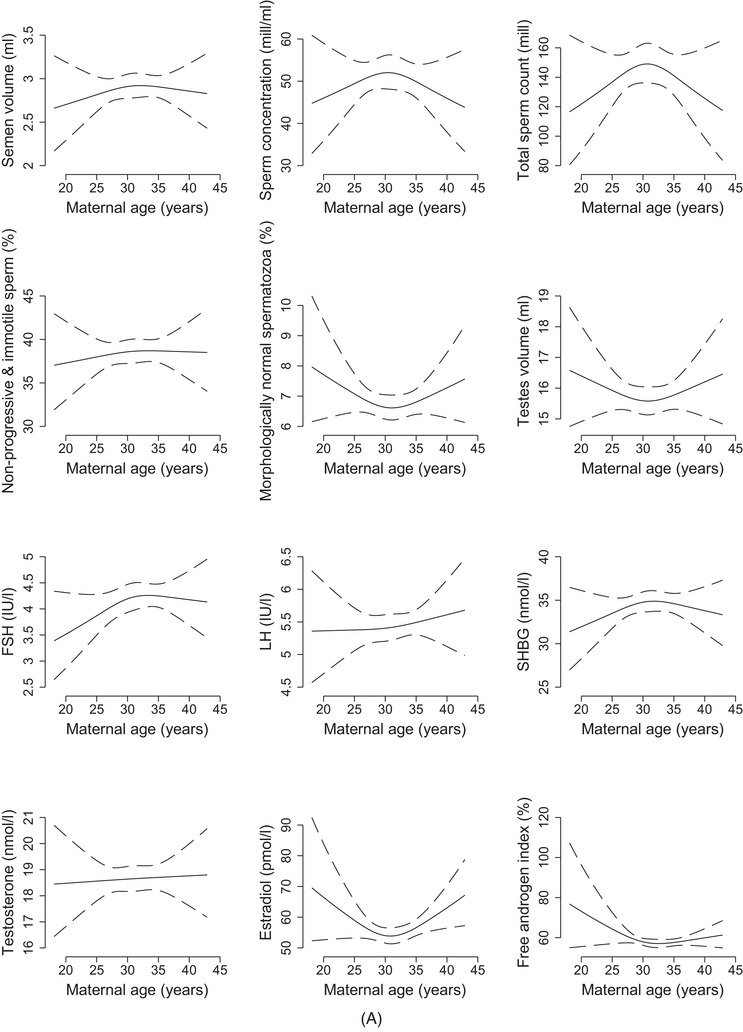

**Figure:**
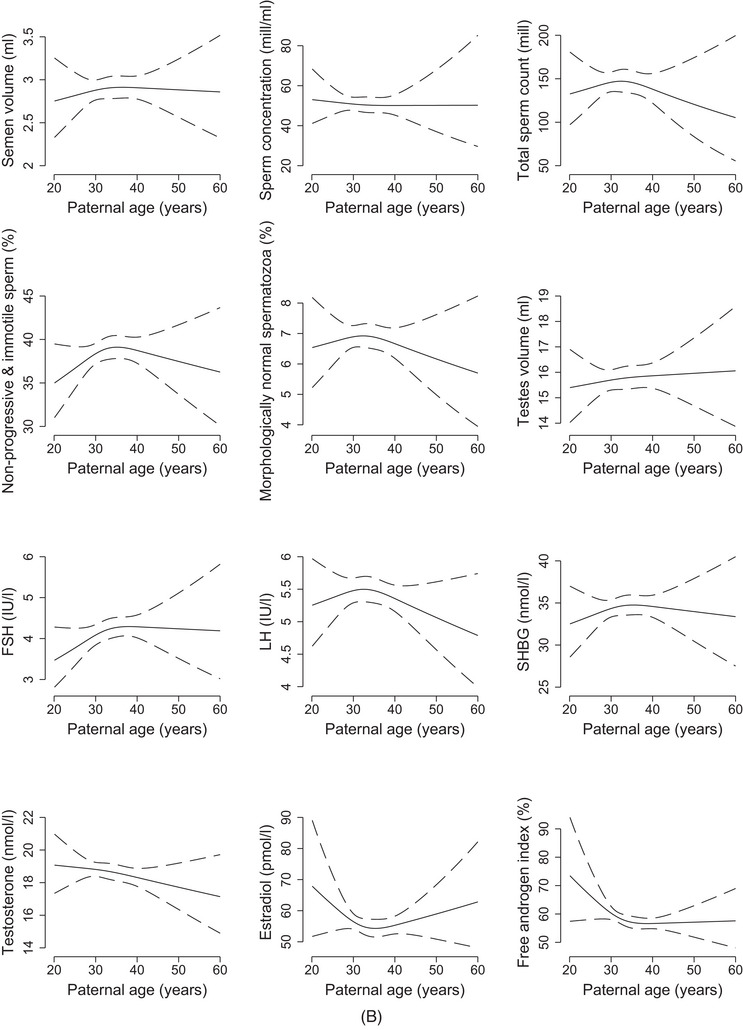

FIGURE 2(A) Adjusted relative difference (in %) in semen characteristics, testis volume and reproductive hormones according to categories of maternal age at birth, FEPOS, Denmark, 1998–2019. Dots representing adjusted estimates with horizontal solid lines representing 95% confidence intervals. (1) Semen volume, sperm concentration, total sperm count and morphology: adjusted for highest parental socioeconomic status, maternal first trimester smoking, maternal pre‐pregnancy BMI, parity, paternal age, place of semen sample collection, abstinence time, and spillage. (2) Motility: adjusted for highest parental socioeconomic status, maternal first trimester smoking, maternal pre‐pregnancy BMI, parity, paternal age, place of semen sample collection, abstinence time, spillage and interval from ejaculation to analysis. (3) Testis volume: adjusted for highest parental socioeconomic status, maternal first trimester smoking, maternal pre‐pregnancy BMI, parity, paternal age, and abstinence time. (4) Reproductive hormones: adjusted for highest parental socioeconomic status, maternal first trimester smoking, maternal pre‐pregnancy body mass index, parity, paternal age, and time at blood sample collection. * We excluded participants with (1) spillage at collection from the analyses of semen volume and total sperm count (*n* = 182), and (2) azoospermia from the analyses of motility and morphology (*n* = 17). (B) Adjusted relative difference (in %) in semen characteristics, testis volume and reproductive hormones according to categories of paternal age at birth, FEPOS, Denmark, 1998–2019. Dots representing adjusted estimates with horizontal solid lines representing 95% confidence intervals.Abbreviations: FSH, follicle stimulating hormone; LH, luteinizing hormone; SHBG, sex‐hormone binding globulin. (1) Semen volume, sperm concentration, total sperm count and morphology: adjusted for highest parental socioeconomic status, paternal smoking, maternal age, place of semen sample collection, abstinence time, and spillage. (2) Motility: sdjusted for highest parental socioeconomic status, paternal smoking, maternal age, place of semen sample collection, abstinence time, spillage and interval from ejaculation to analysis. (3) Testis volume: adjusted for highest parental socioeconomic status, paternal smoking, maternal age, and abstinence time. (4) Reproductive hormones: adjusted for highest parental socioeconomic status, paternal smoking, maternal age and time at blood sample collection. * We excluded participants with (1) spillage at collection from the analyses of semen volume and total sperm count (*n* = 182), and (2) azoospermia from the analyses of motility and morphology (*n* = 17).

**Figure:**
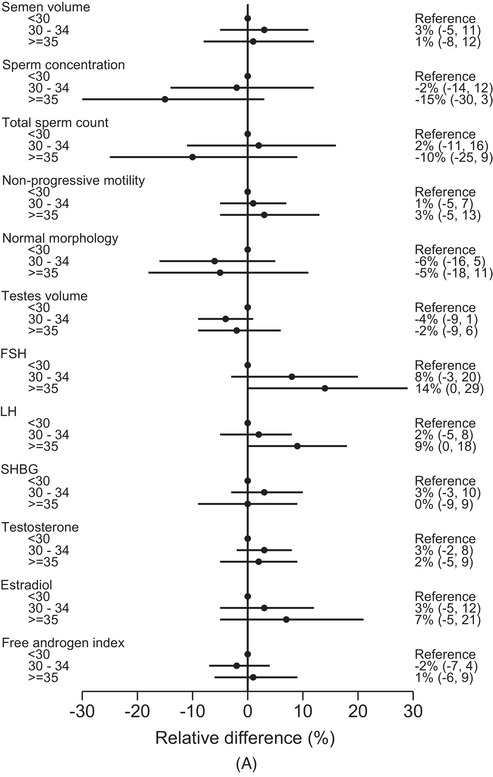

**Figure:**
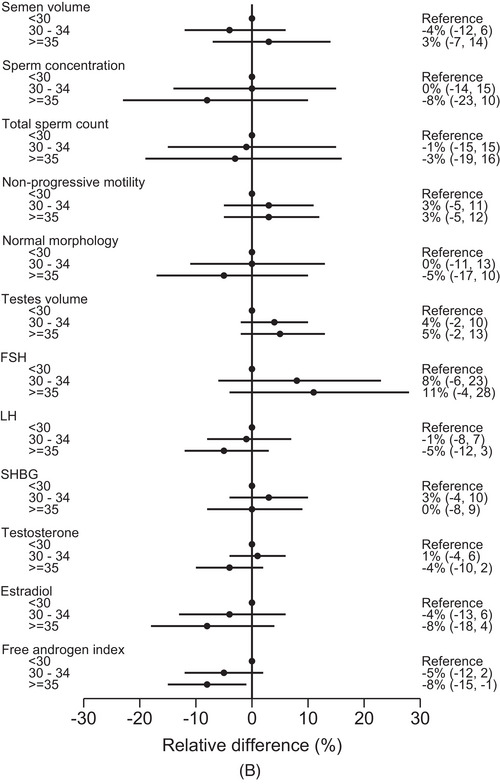

### 3.2 Main analyses: paternal age

In the spline plots, deviations across the age span were less distinct than for maternal age, except for testosterone showing a downward tendency with higher age (Figure 1B). To note, the maternal and paternal curves were relatively flat within the age spans representing the majority of our study population and with broad confidence intervals at the ends of the spectrums. In the adjusted analyses, the findings according to paternal age categories were not consistent across the outcomes (Figure 2B and Table S3). In comparison to the reference, sons of fathers ≥ 35 years had slightly lower sperm concentration (−8% (95% CI: −23, 10)) but higher testis volume (5% (95% CI: −2, 13)) (Figure 2B). FSH was higher (11% (95 CI%: −4, 28)), whereas testosterone (−4% (95% CI: −10, 2)) and estradiol (−8% (95% CI: −18, 4)) were slightly lower in sons of fathers ≥ 35 years. In general, the confidence intervals were quite broad.

### 3.3 Sensitivity analyses

We did not observe specific patterns in the sensitivity analysis with further categorization of paternal age, although the proportion of morphologically normal sperm cells as well as the level of LH, estradiol, and FAI was lower in sons of fathers > 45 years (Figure S2). In the analysis with parental age combinations, we found an indication of lower sperm concentration for all of the age combinations when compared to the reference where both parents were < 30 years of age. However, the results did not support a dose–response. Further, some cells were with limited numbers leading to very wide confidence why results should be interpreted cautiously (Table S4). In particular, sperm concentration for the oldest age combinations was lower, that is, both mother and father ≥ 35 years, *n* = 144 (−27%, 95% CI: −40, −19).

## 4 DISCUSSION

### 4.1 Principal findings

In this large population‐based cohort study, we did not observe strong associations between higher parental age at birth and biomarkers of fecundity in their young adult sons. However, we cannot exclude poorer semen characteristics, especially sperm concentration, in sons born by older mothers (≥ 35 years) or high age of both parents. In sons of mothers ≥ 35 years, the possible impaired testicular function was supported by findings of higher concentrations of FSH and LH but not lower testosterone.

### 4.2 Previous literature

Only few studies have been published on the association between parental age at birth and reproductive health in male offspring. In 2014, a Danish register‐based study examined the association with semen characteristics in a cohort of 10,965 men referred for semen analysis due to partnership infertility.^20^ The results for semen characteristics obtained in our study are in line with theirs, although the studies are not directly comparable. In their study, the population was large and with wide contrast in parental age, while we performed standardized and quality‐controlled semen analyses for research purposes, used a population‐based sample and was able to perform important adjustments. In our study, we also studied other aspect of fecundity, showing modest, if any, associations.

Overall, our sensitivity analysis with parental age combinations indicated lower sperm concentration for the combinations, where either one or both of the parents were of high age, compared to the reference, where both parents were < 30 years. However, the analysis did indicate a specific dose‐dependent association, although the diagonal line going from the reference through both parents 30–34 years (*n* = 213) to both parents ≥ 35 years (*n* = 144) showed increasingly lower sperm concentration with higher age of the parents. Further, the analysis was limited by low numbers of individuals especially for some of the age combinations, leading to very wide confidence intervals. Finally, we might not have captured confounding for specific age constellations such, as father ≥35 years and mother < 30 years, driven by unknown socioeconomic or behavioral patterns. We therefore urge for cautious interpretation of these findings. In the sensitivity analysis with fathers of higher age (> 45 years), we did not detect any strong associations except for morphology.

### 4.3 Strengths and limitations

The main strengths of the study include the large study population, longitudinal design, close to complete exposure information, measurements of multiple biomarkers of fecundity in a uniform age group, semen analysis according to a standard protocol with external quality assurance program, and detailed information on important confounding and precision variables. In the maternal main analysis, confounder adjustment pulled the estimates in a more unfavorable direction for the semen characteristics, especially for sperm concentration and total sperm count, and for some reproductive hormone concentrations like FSH, LH, and estradiol (Table S3). In the paternal main analysis, the estimates were less affected by the confounder adjustment. We cannot, however, exclude residual confounding or confounding from unknown factors.

However, we need to consider some limitations. Data on parental age was extracted from a register with virtually no measurement error. Still, we cannot guarantee that the father registered at the birth certificate is in fact the biological father. The proportion of non‐paternity has been reported to be low,^24^ and we do believe that any misclassification is random and not depending on the age of the father. An additional limitation was the lack of parents at the end of the age spectrums. Hereby, the study is not informative about possible effects of maternal age in the upper part of the reproductive window and paternal age above 40 years. The assessment of semen characteristics and reproductive hormones was conducted by two trained and blinded biomedical laboratory technicians. Still, the use of a single blood and semen sample per individual inevitably introduces measurement error. Semen characteristics show wide intra‐individual, between‐day variation; however, earlier studies report that any error using a single sample to determine semen characteristics would most likely be random.^25^ Reproductive hormone concentrations show intra‐individual variability and for especially testosterone diurnal variation.^26^, ^27^, ^28^ Diurnal variation was sought handled by adjusting for timing of blood sampling. We cannot exclude that intra‐individual variability might have affected our results, however, most likely with random error. Although fecundity was not directly measured in our study, semen quality and reproductive hormone concentrations are considered important markers of male fecundity.^3^, ^4^ The participation rate in FEPOS was low (19%) as seen in most studies on semen quality.^29^ In a recent study, we investigated the impact of potential differential participation in FEPOS showing limited risk of selection bias that only introduced small changes in estimates in etiologic studies.^30^ Further, as the majority of men at young ages are probably unaware of their fecundity, we believe that participation was unrelated to their reproductive health. Finally, pre‐specified selection weights were applied in our analyses.

It is a potential drawback that the results were not uniform across the semen characteristics, however, only if we assume that parental age affects fecundity universally. On the other hand, parental age at birth could instead affect only single or groups of biomarkers specifically. In sons of mothers ≥ 35 years, testosterone concentrations were comparable to the reference group. We would expect testosterone concentrations to be lower if the findings for the semen characteristics signaled impaired testicular function. However, LH and FSH concentrations were higher in the group of sons of mothers ≥ 35 years which could be compatible with impaired spermatogenesis as indicated by lower sperm concentration and total sperm count. In earlier studies, significantly higher levels of FSH and LH have been shown in a cohort of infertile compared to fertile men, and negative correlations between FSH, LH and semen characteristics have been reported in a large Australian birth cohort of young men,^31^, ^32^ providing potential links between our findings for the different biomarkers. Still, we cannot exclude that our findings occurred by chance due to testing of multiple outcomes.

## 5 CONCLUSION

We found no strong association between higher parental age at birth and biomarkers of fecundity in young men. We, therefore, find it unlikely that the high proportion of men with poor semen quality and fecundity in recent generations has been influenced by parental decision to delay childbearing. Further studies including a broader parental age range and repeated measures of reproductive health in their sons are needed to explore the potential signals that we observed for the upper part of the parental age distribution.

## AUTHOR CONTRIBUTIONS

*Study conception and design*: AE, SST, GT, JPB, CHRH. *Acquisition of data*: SST, KSH, JPB, CHRH. *Statistical analysis*: AE. *Interpretation of data*: AE, NB, AGS, LHA, SST, KSH, GT, JPB, CHRH. *Drafting of the article*: AE. *Critical revision of the article for important intellectual content*: all. *Final approval of the version to be published*: all.

## FUNDING INFORMATION

the ReproUnion collaborative study, co‐financed by the European Union, Intereg V ÖKS (20200407); the Lundbeck Foundation (R170‐2014‐855); the Capital Region of Denmark, Region Skåne, and The Medical Faculty at Lund University, Sweden, Medical doctor Sofus Carl Emil Friis and spouse Olga Doris Friis's Grant, Axel Muusfeldt's Foundation (2016‐491); AP Møller Foundation (16‐37), the Health Foundation and Dagmar Marshall's Fond; Aarhus University Forskningsfond and Independent Research Fund Denmark (9039‐00128B); the European Union (ERC, BIOSFER, 101071773)

## CONFLICT OF INTEREST STATEMENT

Sandra S. Tøttenborg (SST) serve as guest editor of the special issue “Periconceptional paternal exposures, pregnancy outcomes and health of the offspring” that this paper is submitted to. By agreement with the Andrology editorial team, SST will recuse herself from any editorial decisions regarding this paper.

## Supporting information

Supporting Information

## Data Availability

The data are not publicly available due to privacy or ethical restrictions. However, access to data can be applied for through the procedure described at https://www.dnbc.dk/access‐to‐dnbc‐data.
