# Supplementary material for: Parental age at birth and biomarkers of fecundity in young Danish men
Source: Andrology. 2023 Sep 26;13(1):89–100. doi: 10.1111/andr.13536 (PMC11635595; doi:10.1111/andr.13536)
Supplement: Supplementary file 1 — Supporting Information [file ANDR-13-89-s001.docx]

**SUPPLEMENTARY MATERIALE**

**TITLE**: Parental age at birth and biomarkers of fecundity in young Danish men

Figures

**Supplementary Figure 1.** Flowchart of participants in the study, FEPOS, Denmark, 1998-2019.

Eligible for sampling to FEPOS

(n=21,623)

Sampled and invited to FEPOS

(n=5,697)

Completed FEPOS questionnaire

(n=1,173)

Not sampled

(n=15,927)

Declined participation or never responded

(n=4,524)

Participated in clinical examination

(n=1,057)

Did not complete clinical examination or no information on maternal age

(n=116)

Information on parental age available

Maternal: n=1,057

Paternal: n=1,048

Information on paternal age missing:

Paternal: n=9

**Supplementary Figure 2.**

Adjusted relative difference (in %) in semen characteristics, testis volume and reproductive hormones according to further categorization of paternal age at birth, FEPOS, Denmark, 1998–2019. Dots representing adjusted estimates with horizontal solid lines representing 95% confidence intervals.

Abbreviations: FSH, follicle stimulating hormone; LH, luteinizing hormone; SHBG, sex-hormone binding globulin.

1) Semen volume, sperm concentration, total sperm count and morphology: Adjusted for highest parental socioeconomic status, paternal smoking, maternal age, place of semen sample collection, abstinence time, and spillage.

2) Motility: Adjusted for highest parental socioeconomic status, paternal smoking, maternal age, place of semen sample collection, abstinence time, spillage and interval from ejaculation to analysis.

3) Testis volume: Adjusted for highest parental socioeconomic status, paternal smoking, maternal age, and abstinence time.

4) Reproductive hormones: Adjusted for highest parental socioeconomic status, paternal smoking, maternal age and time at blood sample collection.

* We excluded participants with 1) spillage at collection from the analyses of semen volume and total sperm count (n=182), and 2) azoospermia from the analyses of motility and morphology (n=17)


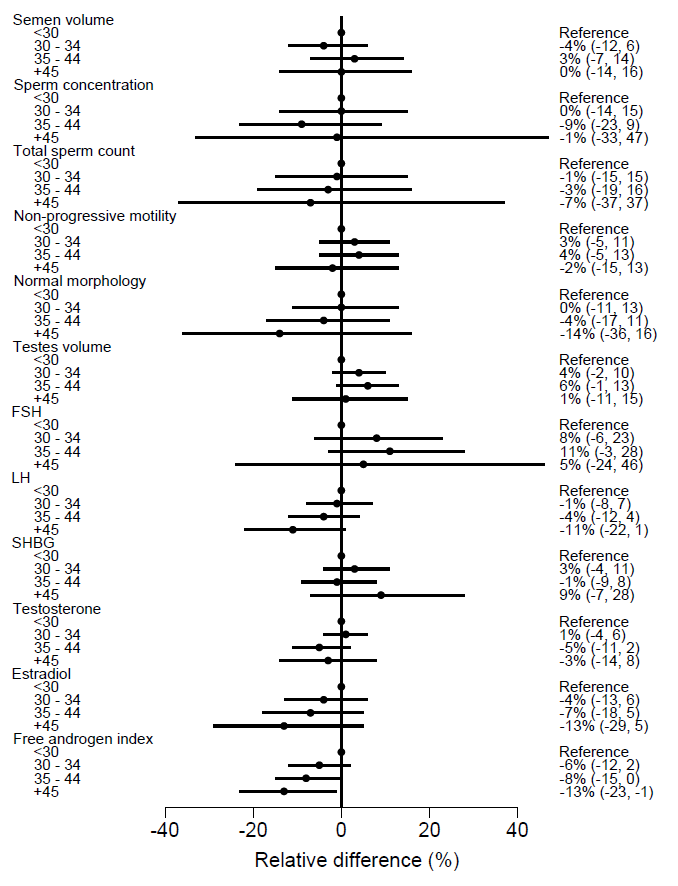


Tables

**Supplementary Table 1**. Baseline characteristics according to categories of paternal age at birth, n=1048, FEPOS, Denmark, 1998 – 2019.

|  | <30 years^a^  N = 290 | 30 – 34 years  N = 391 | ≥ 35 years  N = 367 | Missings, N |
| --- | --- | --- | --- | --- |
| Maternal pre-pregnancy BMI (kg/m2), n (%)^b^ |  |  |  | 24 |
| Underweight | 16 (5.7) | 26 (6.8) | 20 (5.6) |  |
| Normal | 201 (71.3) | 271 (70.4) | 280 (78.4) |  |
| Overweight | 49 (17.4) | 69 (17.9) | 45 (12.6) |  |
| Obese | 16 (5.7) | 19 (4.9) | 12 (3.4) |  |
| Maternal smoking during 1. trimester, n (%) |  |  |  | 0 |
| Non-smoker | 210 (72.4) | 304 (77.7) | 296 (80.7) |  |
| 0-10 cigarettes per day | 65 (22.4) | 75 (19.2) | 60 (16.3) |  |
| >10 cigarettes per day | 15 (5.2) | 12 (3.1) | 11 (3.0) |  |
| Maternal age at birth (years), mean (SD) | 27.4 (3.1) | 30.9 (3.0) | 33.9 (3.6) | 0 |
| Paternal age at birth (years), mean (SD) | 27.3 (2.0) | 32.3 (1.4) | 39.3 (4.2) | 0 |
| Paternal smoking, n (%) |  |  |  | 0 |
| No | 208 (71.4) | 281 (72.1) | 253 (68.9) |  |
| Yes | 83 (28.6) | 109 (27.9) | 114 (31.1) |  |
| Parity, n (%) |  |  |  | 21 |
| 1. child | 194 (68.3) | 156 (40.9) | 110 (30.4) |  |
| 2. or more child | 90 (31.7) | 225 (59.1) | 252 (69.6) |  |
| Highest parental social class, n (%) |  |  |  | 0 |
| High grade professional | 53 (18.3) | 150 (38.4) | <155 (42.2) |  |
| Low grade professional | 90 (31.0) | 125 (32.0) | 133 (36.2) |  |
| Skilled or unskilled worker | 111 (38.3) | 108 (27.6) | 76 (20.7) |  |
| Student or economically inactive | 36 (12.4) | 8 (2.0) | < 5 (.) |  |
| Time to pregnancy, n (%) |  |  |  | 6 |
| <6 months | <190 (<65.5) | <249 (<63.7) | <195 (<53.1) |  |
| 6 – 12 months | 27 (9.4) | 40 (10.3) | 39 (10.7) |  |
| >12 months or MAR | 20 (6.9) | 50 (12.8) | 68 (18.7) |  |
| Unplanned | 53 (18.4) | 52 (13.3) | 65 (17.9) |  |
| Place of semen sample, n (%) |  |  |  | 10 |
| Home | 39 (13.6) | 50 (12.9) | 49 (13.5) |  |
| Clinic | <251 (86.6) | <341 (87.2) | <318 (86.6) |  |
| Abstinence time, n (%) |  |  |  | 5 |
| <2 days | 92 (31.7) | 139 (35.7) | 129 (35.4) |  |
| 2-3 days | 136 (46.9) | <186 (47.6) | <170 (46.3) |  |
| >3 days | 62 (21.4) | 66 (17.0) | 68 (18.7) |  |
| Spillage, n (%) |  |  |  | 9 |
| No | <249 (85.9) | <323 (82.6) | <297 (80.9) |  |
| Yes | 41 (14.2) | 68 (17.6) | 70 (19.3) |  |
| Interval from ejaculation to analysis, n (%) |  |  |  | 12 |
| 0-60 minutes | 236 (82.8) | <331 (84.7) | <305 (83.1) |  |
| >60 minutes | 49 (17.2) | 60 (15.5) | 62 (17.1) |  |
| Time blood sampling, n (%) |  |  |  | 10 |
| Morning <12 PM | 112 (39.2) | 150 (38.8) | 112 (30.7) |  |
| Afternoon 12-18 PM | <154 (53.1) | <195 (49.9) | <215 (58.6) |  |
| Evening >18 PM | 24 (8.4) | 46 (11.9) | 40 (11.0) |  |

Abbreviations: AAM, age at menarche; BMI, body-mass index; MAR, medically assisted reproduction. ^a^ Due to local data regulations, it is not allowed to report smaller numbers than five. Thus, numbers have been changed to mask the numbers smaller than five.

The percentage distribution for the levels of each specific baseline characteristic within each age category.

|  |  | Total |
| --- | --- | --- |
|  | Median (25th – 75th pseudo percentile)^a^ | N |
| **Semen characteristics** |  |  |
| Semen volume (ml) | 2.7 (1.9; 3.6) | 865 |
| Sperm concentration (million/ml) | 38.6 (18.8; 72.2) | 1051 |
| Total sperm count (million) | 102.5 (45.4; 199.4) | 865 |
| Non-progressive and immotile spermatozoa (%) | 37.0 (26.0; 47.8) | 1034 |
| Morphologically normal sperm (%) | 6.0 (3.0; 10.0) | 1028 |
| **Testes volume** |  |  |
| Average testis volume (ml) | 15.0 (12.0; 20.0) | 1054 |
| **Reproductive Hormones** |  |  |
| FSH (IU/L) | 3.5 (2.5; 5.1) | 1044 |
| LH (IU/L) | 5.1 (4.0; 6.6) | 1044 |
| SHBG (nmol/L) | 33.0 (25.0; 41.0) | 1044 |
| Estradiol (pmol/L) | 52.2 (34.6; 73.2) | 1045 |
| Testosterone (nmol/L) | 18.1 (14.8; 22.0) | 1045 |
| FAI^b^ | 55.5 (45.3; 68.8) | 1044 |

**Supplementary Table 2.** Crude distribution of semen characteristics, testis volume and reproductive hormones, n=1057, FEPOS, Denmark, 1998–2019.

Abbreviations: FSH, follicle-stimulation hormone; LH, luteinizing hormones; SHGB, sex hormone binding globulin; FAI, free androgen index
^a^ Pseudo percentiles were calculated as the mean of the five values nearest to the actual percentile to comply with local data regulations stating that a single value corresponding to a single participant cannot be reported. ^b^ Calculated as FAI = (testosterone/SHGB)*100

**Supplementary Table 3**. Crude and adjusted relative difference (in %) in semen characteristics, testis volume and reproductive hormones according to categories of maternal and paternal age at birth, FEPOS, Denmark, 1998–2019.

|  | Maternal age categories | | | | |
| --- | --- | --- | --- | --- | --- |
|  | < 30 years | 30 – 34 years | | ≥ 35 years | |
| **Outcome** |  | Unadjusted | Adjusted | Unadjusted | Adjusted |
| Semen volume^a,e^ | Ref. | 3% (-4, 12) | 3% (-5, 11) | 2% (-6, 12) | 1% (-8, 12) |
| Sperm concentration^a^ | Ref. | 8% (-4, 22) | -2% (-14, 12) | -10% (-23, 5) | -15% (-30, 3) |
| Total sperm count^a, e^ | Ref. | 11% (-4, 27) | 2% (-11, 16) | -6% (-23, 15) | -10% (-25, 9) |
| Non-progressive motility^b, e^ | Ref. | 2% (-4, 8) | 1% (-5, 7) | 3 (-4, 11) | 3% ( -5, 13) |
| Normal morphology^a, e^ | Ref. | -4% (-13, 6) | -6% (-16, 5) | -2% (-14, 11) | -5% (-18, 11) |
| Testis volume^c^ | Ref. | -2% (-6, 3) | -4% (-9, 1) | 0% (-6, 7) | -2% (-9, 6) |
| FSH^d^ | Ref. | 5% (-6, 17) | 8% (-3, 20) | 10% (-3, 24) | 14% (0, 29) |
| LH^d^ | Ref. | 0% (-6, 6) | 2% (-5, 8) | 4% (-3, 13) | 9% (0, 18) |
| SHBG^d^ | Ref. | 3% (-3, 9) | 3% (-3, 10) | 1% (-6, 8) | 0% (-9, 9) |
| Testosterone^d^ | Ref. | 2% (-3, 6) | 3% (-2, 8) | -1% (-6, 5) | 2% (-5, 9) |
| Estradiol^d^ | Ref. | -1% (-9, 8) | 3% (-5, 12) | 1% (-9, 12) | 7% (-5, 21) |
| Free androgen index^d^ | Ref. | -4% (-11, 3) | -2% (-7, 4) | -5% (-13, 2) | 1% (-6, 9) |
|  |  |  |  |  |  |
|  | Paternal age categories | | | | |
|  | < 30 years | 30 – 34 years | | ≥ 35 years | |
| **Outcome** |  | Unadjusted | Adjusted | Unadjusted | Adjusted |
| Semen volume^a,e^ | Ref. | -3% (-11, 5) | -4% (-12, 6) | 2% (-6, 11) | 3% (-7, 14) |
| Sperm concentration^a^ | Ref. | 1% (-12, 15) | 0% (-14, 15) | -5% (-17, 10) | -8% (-23, 10) |
| Total sperm count^a, e^ | Ref. | 3% (-12, 20) | -1% (-15, 15) | -2% (-17, 15) | -3% (-19, 16) |
| Non-progressive motility^b, e^ | Ref. | 3% (-3, 10) | 3% (-5, 11) | 3% (-4, 10) | 3% (-5, 12) |
| Normal morphology^a, e^ | Ref. | 0% (-10, 12) | 0% (-11, 13) | -3% (-14, 9) | -5% (-17, 10) |
| Testis volume^c^ | Ref. | 3% (-2, 9) | 4% (-2, 10) | 3% (-2, 9) | 5% (-2, 13) |
| FSH^d^ | Ref. | 7% (-7, 23) | 8% (-6, 23) | 10% (-4, 27) | 11% (-4, 28) |
| LH^d^ | Ref. | 0% (-7, 8) | -1% (-8, 7) | -2% (-9, 6) | -5% (-12, 3) |
| SHBG^d^ | Ref. | 3% (-3, 10) | 3% (-4, 10) | 0% (-6, 7) | 0% (-8, 9) |
| Testosterone^d^ | Ref. | 1% (-4, 6) | 1% (-4, 6) | -3% (-8, 2) | -4% (-10, 2) |
| Estradiol^d^ | Ref. | -2% (-12, 8) | -4% (-13, 6) | -4% (-13, 7) | -8% (-18, 4) |
| Free androgen index^d^ | Ref. | -7% (-15, 3) | -5% (-12, 2) | -10% (-18, -1) | -8% (-15, -1) |

Abbreviations: FSH, follicle stimulating hormone; LH, luteinizing hormone; SHBG, sex-hormone binding globulin.

^a^ Semen volume, sperm concentration, total sperm count and morphology: Adjusted for highest parental socioeconomic status, maternal first trimester smoking, maternal pre-pregnancy body mass index, parity, paternal age, place of semen sample collection, abstinence time, and spillage.

^b^ Motility: Adjusted for highest parental socioeconomic status, maternal first trimester smoking, maternal pre-pregnancy body mass index, parity, paternal age, place of semen sample collection, abstinence time, spillage and interval from ejaculation to analysis.

^c^ Testis volume: Adjusted for highest parental socioeconomic status, maternal first trimester smoking, maternal pre-pregnancy body mass index, parity, paternal age, and abstinence time.

^d^ Reproductive hormones: Adjusted for highest parental socioeconomic status, maternal first trimester smoking, maternal pre-pregnancy body mass index, parity, paternal age, and time at blood sample collection.

^e^ We excluded participants with 1) spillage at collection from the analyses of semen volume and total sperm count (n=182), and 2) azoospermia from the analyses of motility and morphology (n=17)

**Supplementary Table 4.** Adjusted relative difference (in %) for sperm concentration according to parental age combinations, FEPOS, Denmark, 1998-2019.

|  |  | **Paternal age categories (years)** | | |
| --- | --- | --- | --- | --- |
|  |  | <30 | 30 – 34 | >=35 |
| **Maternal age categories (years)** | <30 | Reference (n=243) | -13% (-27, 4) (n=145) | -26% (-45, 1)  (n=52) |
|  | 30 - 34 | -20% (-39, 3) (n=40) | -12% (-26, 3) (n=213) | -9% (-24, 9) (n=171) |
|  | >=35 | -67% (-82, -38)  (n=7) | -3% (-29, 32) (n=33) | -27% (-40, -19)  (n=144) |
